# Return of health research findings in Nigeria-perspective of potential research participants

**DOI:** 10.21203/rs.3.rs-7553414/v1

**Published:** 2025-10-22

**Authors:** Victor Chukwukelu, Onochie Okoye, Elias Aniwada

**Affiliations:** College of Medicine,University of Nigeria; College of Medicine,University of Nigeria; College of Medicine,University of Nigeria

**Keywords:** Health Research, Research Results, Incidental Findings, Research Participants, Perspectives, Nigeria

## Abstract

**Background:**

Health research in Nigeria, a low- and middle-income country with significant genetic diversity and disease burden, raises ethical challenges regarding the return of individual research findings to participants. Though there is a growing body of literature on participants’ perspectives regarding these issues globally, there remains a paucity of empirical evidence emanating from Nigeria. This study explored the perspectives of potential research participants in Nigeria on the return of primary research results (RRs) and incidental findings (IFs), especially from human biological specimen (HBS)-based research

**Methods:**

Using a mixed-methods approach (quantitative and qualitative methods), a cross-sectional survey of 405 urban residents and focus group discussions involving 4 groups (gender/age stratified) with 30 participants were conducted within Enugu metropolis of Nigeria in 2021. The quantitative data was collected using a semi-structured, interviewer-administered questionnaire, and analyzed using Statistical Package for Social Science software program (IBM SPSS v. 20 Inc., Chicago II, USA). Researchers used descriptive statistics (frequencies and percentages) and chi-square tests to find connections between participants’ characteristics and their perspective on having research findings returned to them. A p-value of less than 0.05 was considered statistically significant. Manual thematic content analysis was used for the qualitative data.

**Results:**

Most respondents (97%) desired the return of individual results, prioritizing actionable findings with clinical utility. Many struggled to distinguish between RRs and IFs, indicating a potential misunderstanding of nature and limitations of different research findings. The study revealed a strong trust in researchers but also highlighted misconceptions about the distinction between health research and healthcare. Preferences for in-person disclosure were driven by concerns over confidentiality. Key barriers included inadequate funding, logistical challenges, and low health literacy. Participants recommended measures such as increased funding, training for researchers, and culturally sensitive policies to address these challenges.

**Conclusion:**

The findings underscore strong community demand for the return of individual research results, especially clinically actionable information, but also reveal critical gaps in understanding, resources, and infrastructure. Addressing misconceptions, safeguarding confidentiality, and implementing culturally sensitive, well-funded strategies are essential to ensure ethical and effective return of results in health research.

## Background

Nigeria, as the most populous black nation on earth, and characterized by strong family and community ties in its diverse, heterogenous society, is fast becoming a prominent site for health research due to its high disease burden and genetic diversity(1). Health research involving human research participants and their biological specimens, especially against the backdrop of globalization of research and intensification of genomics/genetics research, raises particularly challenging questions on the researchers’ obligations regarding disclosure of research findings, including whether and under what circumstances to return research results and other findings to participants, their families, and communities(2, 3, 4, 5, 6, 7). These studies, which may include clinical trials, genomic research, epidemiological studies, or other biomedical and socio-behavioral research, have the potential to generate a vast amount of pertinent research results and other findings- incidental and secondary findings-making these ethical, practical and regulatory questions of their return more urgent than ever.

The practice of returning individual primary research results(the general pertinent findings which are related to the main research questions and objectives of a study) and incidental findings(anticipatable/unanticipatable findings that are outside the primary aim of a research study but may hold clinical or personal significance for the participant) is not a novel concept(8), but its implementation in Low and Middle-Income Countries (LMICs) including Nigeria, is fraught with challenges; with the decisions on return of findings often being made by the policy makers, research ethics committees and the researchers with little or no consideration for the voices and preferences of the research participants themselves (9). In these countries, these issues gain greater prominence due to the combination of inadequate health system infrastructures, deficiencies in research governance, varied cultural contexts, and the research participants’ anticipation of direct tangible benefits from their involvement in research(10). For instance, it is uncertain whether the public comprehends the nature and limitations of these findings and whether they would prefer information that has minimal or no clinical value. A major ethical issue is deciding what research findings to share with participants. Should researchers disclose everything, including information that isn’t clinically certain or could cause a participant significant distress without offering a clear treatment plan? There is also the question of who provides follow-up care, especially in a country with a fragile healthcare system. The financial and logistical costs of sharing results and arranging care are often substantial and typically not covered by research funding(3, 4, 11, 12).

While international guidelines and national regulations exist, they often fail to capture the unique cultural contexts and perspectives of research participants themselves(13, 14, 15, 16, 17). National guidelines for human subjects’ research in Nigeria offer broad principles–respect for persons, beneficence, justice–but provide little specific guidance on returning results or incidental findings(18, 19). This knowledge gap creates uncertainty for researchers and leaves participants’ expectations unclear, which can lead to a mismatch between the guidelines and what happens during the research. While ethics committees in Nigeria and across Africa increasingly review protocols for compliance with international standards, there is limited empirical research on how participants themselves view the return of primary research results and incidental findings (20). Without understanding the perspectives of the people most directly affected by these decisions, ethical guidelines and practices will remain incomplete, potentially ineffective, and disconnected from local and cultural realities(21, 22). Lacking real data on what research participants think creates a major problem for ethics and policy. Without this information, researchers and ethics committees cannot make decisions that fit the local context. Instead of reflecting local values, guidelines might just mirror outside priorities, which could weaken ethical standards and erode participant trust(23).

Though there is a growing body of literature on participants’ perspectives regarding these issues, there remains a paucity of empirical evidence emanating from Nigeria(24, 25, 26, 27, 28, 29). Much of the existing literature reflects the perspectives of researchers, ethicists, ethics committee members, and policymakers rather than those of potential participants(30). In general, much of the research on participants’ attitudes makes comparisons and conclusions based on race and ethnicity(31). When conducting health research, it is crucial to prioritize the perspectives of these participants. Their reasons for joining a study, what they hope to gain, and their personal values all influence their willingness to participate. Ethically, they should also have a say in decisions that impact their health. Ignoring their viewpoints can lead to a deeper sense of mistrust, especially in communities that have already experienced historical exploitation or misinformation. Understanding what participants think and feel can help researchers anticipate potential challenges, create culturally sensitive consent forms, and communicate more effectively. Furthermore, the insights gained from Nigerian participants could also be valuable for research in other similar low- and middle-income countries.

In seeking to contribute to a more just and ethical framework for health research in Nigeria and beyond, our paper explores this critical, yet under-explored, under-represented and under-reported aspect of health research by providing a nuanced, potential participant-centered perspective on the return of health research findings, especially from human biological specimen (HBS)-based research. By examining the views, expectations, and concerns of potential research participants in Enugu State of Nigeria, it aims to contribute to the discourse on the development of ethically sound, culturally sensitive, and practically feasible approaches to the disclosure of primary research results and incidental findings in Nigeria.

## Materials and Methods

Our study adopted a mixed-methods approach in 2021 to determine what residents in Enugu metropolis of Nigeria think about having their health research findings returned to them. Enugu metropolis of Enugu State, comprising Enugu North, Enugu South and Enugu East local government areas(LGAs), is one of the most densely populated cities in Nigeria, with 996,481 as the 2017 projected population(32). It is located between Lat. 06°, 26’ and 06°, 30’ N and Long. 07°, 27’ and 07°, 37’ E and lies east of Niger Delta region of Nigeria. The area is predominantly populated by Igbo-speaking Nigerians, many of whom are public servants, professionals, students, artisans, and traders/businesspersons. The metropolis is host to at least 4 tertiary health institutions and 8 tertiary education institutions. Our research had two parts: a cross-sectional quantitative survey and a qualitative study using focus group discussions (FGD). A sequential explanatory design was used. All the interviews were either conducted in English language or Igbo Language, according to the preferences of the participants. The questionnaire for the quantitative component (an additional file shows this in more detail-See additional file1) and the discussion guide used for the FGDs (an additional file shows this in more detail-see additional file 2) were developed specifically for this study, following a thorough literature review to identify existing instruments related to our research topic (33, 34, 35, 36, 37, 38, 39, 40), as well as initial interviews with some health researchers (Public health, bioethics, social sciences, laboratory sciences) and lay persons. Modifications were made to suit our study. Three experienced health researchers were further consulted to review and provide feedback to us on the relevance, clarity and comprehensiveness of the questions. The instruments were pre-tested with a small group of participants (15 and 6 urban residents respectively for the questionnaire and FGD guide) similar to our target population, with a view to identifying and resolving any issues with the questions, such as ambiguity or difficulty in understanding. Some community leaders were also consulted to review the instruments to ensure they were culturally sensitive and relevant to our target population. Based on the feedback and results of these actions, we revised and refined the instruments to ensure they were valid and effective.

The main survey was conducted first, with 405 adults aged 18 and older. These participants were selected using a multi-stage random sampling method from nine different neighborhoods across the 3 LGAs constituting the metropolis. The quantitative data was collected using a semi-structured questionnaire and analyzed using Statistical Package for Social Science software program (IBM SPSS v. 20 Inc., Chicago II, USA). The interviewer-administered questionnaire was designed with a background section and 40 closed-ended items in 5 accompanying sections covering the following variables: sociodemographic variables(age, gender, educational level, employment/perceived socio-economic status); health research participation and perception on research and researchers; willingness/desire for return of results and reasons; perception on format or process for feedback on findings; preferences for types of results; challenges to return of results and measures for overcoming such challenges.

The focus groups, stratified based on age and gender, were held afterward to explore topics that the survey did not fully cover. Participants for the focus groups were purposively selected to ensure a diverse representation of people and were divided into four groups: younger men under 50yrs(YM), older men over 50yrs(OM), younger women under 50yrs(YW), and older women over 50yrs(OW). The qualitative data was gathered through audio-recorded focus group discussions, with each lasting between 45 and 60 minutes. A total of four focus group sessions were conducted by the principal researcher (VNC) to uncover in-depth insight into the participant’s views about the research questions. Each focus group comprised 6–8 participants. Saturation was achieved after one round, with no additional insights or themes being elicited. The audio recordings were then transcribed word-for-word and analyzed using manual thematic content analysis. In reporting the quotes from the participants, identifiers based on the group and the participant number were used. For instance, “OW3” indicates an Older Woman, participant 3. One author (VNC) reviewed all transcripts and independently coded meaning units related to the research questions. This step was validated with the two other authors (OIO and ECA) to ensure that the coding label demonstrates participants’ descriptions without losing the underlying meaning. Subsequently, codes were grouped deductively based on their similarities and differences into preexisting categories that were developed when the questionnaire and FGD questions were designed. Inductive and deductive analyses were conducted, with all authors participating in discussions until agreement was reached on how to structure the data.

Both quantitative and qualitative data were collected and analyzed separately within a specified timeframe. Researchers used descriptive statistics (frequencies and percentages) and chi-square tests to find connections between participants’ characteristics and their perspective on having research results and incidental findings returned to them. A p-value of less than 0.05 was considered statistically significant.

This study adhered to the ethical principles outlined in the Declaration of Helsinki and the National Code for Health Research Ethics (NCHRE) in Nigeria. Informed, written consent encompassing details on the study purpose, objectives, procedure, risks and benefits was obtained from all the participants in both phases. Participants’ information and data were treated with strict confidentiality, ensuring that personal details were anonymized. The data were stored and kept on a computer protected with a password and will be discarded at the specified time recommended in the NCHRE.

## RESULTS

### Results from the quantitative component

#### Socio-demographic variables

A total of 405 urban residents from the 3 different Local Government Areas of Enugu metropolis in Nigeria successfully participated in the survey, giving a response rate of 92.6%. Among them, 50.4% were males, and 94.8% had at least secondary school level of formal education. Their ages ranged from 18 years to 87 years, with the mean (SD) age being 35.46±12.54. The majority of participants (72.8%) were employed either in the public or private sector. Findings on the socio-demographic variables are presented in Table 1.

#### Health research participation and perception on research/researchers/research findings

Among the 405 respondents, only 31.6% had participated in HBS-based health research in the past. The majority (77.3%) had good self-reported general knowledge of health research/research findings, but only 46.4% were confident of their level of knowledge. Statistically significant associations were shown between age, education, occupation (*p* < 0.05, *p* < 0.001, *p* < 0.001 respectively) and confidence about their levels of self-reported knowledge of health research/research findings. Many respondents (68.4%) did not know of the significance of the participation of healthy and normal volunteers in biomedical/HBS-based health research. Only 13.1% had a habit of routinely searching for health research-related information. While most of the respondents (67.4%) demonstrated good knowledge of primary research results (RRs) being the expected logical outcome of any health research, 71.2% did not show good knowledge about incidental findings (IFs). With respect to disclosing or explaining research findings to research participants or the study communities, only 56(13.8%) participants felt it is a responsibility that can be handled by any member of the research team, and not necessarily the main researchers. A majority (71.9%) of the respondents expressed their trust in health researchers and the outcome (RRs and IFs) of their research. A statistically significant association (*p* = 0.001) was found between employment status and trust in researchers, with employed individuals more likely to express trust.

#### Perception on return of research findings

All the respondents expected research findings to be disseminated either to the government, public, research sponsors, or the individual research participants, depending on the context. For most respondents, the expectation for research findings dissemination is based on the notion that it constitutes the final, appropriate step of the research process. Most of the respondents (97%) were interested in receiving their individual research results and expressed their desire for such, with 94.1% regarding treatability and perceived utility/actionability of such findings highly as their main reason. Out of these 393 respondents who desired return of research findings, 189(48.1%) expressed desire to have only the primary research results returned and 204 (51.9%) desired to have both the primary research results and the incidental findings. In determining whether to return individual research findings, the majority (81.2%) of those surveyed believe that the opinions of both the researchers and the research participants should be the most important factor. Table 2 below shows the frequencies of responses concerning the types of research results desired for return.

There was a statistically significant association between respondents’ desire to receive results lacking clinical utility and both age (*p* = 0.005) and educational attainment (*p* = 0.009). Individuals aged 26–35 and those with at least a secondary school education were more inclined to want such results returned. Employment status was significantly associated with the desire to receive results indicating a curable condition (*p* = 0.024), with employed individuals more likely to express this preference. Most of the respondents (73.3%) expressed concerns over confidentiality by indicating preference for return of individual results to be done in-person through a face-to-face interaction; with 88% and 85.4% of them being averse to having results returned by email and phone call respectively. Regarding disclosure of individual results in a public forum, 96% were not in support. Among the reasons given by the respondents to justify return of individual results, 56% of the respondents cited the anticipated provision of direct health benefit to the participants, families and/or their communities as the highest-ranking reason. This was followed closely by the need to encourage future research participation, as well as a mark of acknowledgement and appreciation of the participant’s contribution to the research. When asked about their preferences for sharing individual research results with others besides themselves in a hypothetical scenario, 46.9% of the participants preferred to share such with spouses. The next most preferred individuals to share with were parents (36.8%), who were then followed by siblings and children in that order. Based on the survey results, most participants-283 people, or 69.9%-preferred to receive their individual research results after the research was complete. A smaller group of 62 individuals (15.3%) chose to receive their results during the research, while the remaining 60(14.8%) had no preference.

Many reasons were stated by the respondents as barriers or challenges to the process of returning individual research results and incidental findings. Table 3 below presents the distribution of the known or perceived barriers/challenges. The commonest reason among the respondents was lack of adequate funding for the research (59.5%).

Among measures recommended by the 405 respondents for effective promotion of the return of individual research findings were the following: Provision of adequate fund for research(55.8% or 226 respondents), adequate training of researchers in communication/community engagement(51.1% or 207), provision of adequate incentive for researchers(28.4% or 115),appropriate policies on return of results(25.7% or 104), and sharing of costs between researchers and participants for returning results(4% or 16). Most respondents-311 (76.8%)-supported making cost-sharing optional for research participants, while 7(1.7%) were against the measure, and 87(21.5%) were ‘not sure’. When respondents were questioned about making researchers bear the entire costs for returning individual research results, 326(80.5%) were in support, 48(11.9%) were against the measure, and 31(7.7%) were ‘not sure’. In the survey, most respondents (50.9%) believed that both the government and researchers should be responsible for implementing and monitoring new measures to promote effective return of individual research results. The remaining participants were more divided:24.2% (98 respondents) felt the government alone should be responsible; 12.6% (51 respondents) felt researchers alone should be responsible; 11.1% (45 respondents) were not sure, and 1.2% (5 respondents) thought the responsibility belonged to community members or research participants.

#### Results from the qualitative component

Thirty participants participated in the 4 focus group discussions. The mean age was 44.2 ± 17.5 years. Sixteen (53.3%) of the participants were females. Many of the FGD participants (80%) had at least secondary school level of education, and with respect to their marital status, 36.7% were single/unmarried while the rest were either married or divorced/separated/ widowed.

Five key themes were derived from the data, highlighting the distinctive and noteworthy ways in which the FGD participants perceived the issue of returning individual research results and incidental findings. These themes encompassed their perception on health research, researchers and research findings; levels of willingness to have results returned to them and the rationales behind willingness or reluctance over return of research findings; types of research findings to be returned with rationales for their preferences; format and process of returning research results; and challenges in the return of research findings and measure to mitigate them. Representative quotes corresponding to the identified themes are presented below.

#### Perception on health research, researchers and research findings-Role of trust

Many participants demonstrated a good basic understanding of health research, and trusted researchers and their research findings. However, the level of trust expressed by a few participants appeared guarded due to negative past experiences. Some equated health research with diagnostic lab tests, so their trust in researchers and their findings was influenced by their past experiences with laboratory workers and test results.

*“Health research is done to learn more about human health and to find better ways to prevent and treat disease, I trust researchers*”. (OW1)

“*Health research is work done by some medical personnel as a team, individuals or institutions to solve a particular health puzzle. Findings are sometimes not factual because participants may give wrong information or samples; some subjects may not qualify for research but lie to be included in the research for anticipated benefits. Some researchers are doing well and some others cut corners*”. (YW7)

‘*‘Every research should have a finding, either negative or positive, useful or useless, expected or unexpected, straight-forward or complicated. There may be many findings from single research. The researcher will need to guide the participant* “(YM2)

“*Research is good but because of poverty many people don’t go for check up (lab diagnosis)*”(OM5)

“*Death rate is on the increase because of improper diagnosis (unreliable results) from diagnostic labs*” (OM6).

#### Levels of willingness to have results returned to them as well as the associated rationale

Practically, all the participants were interested in their individual research results and incidental findings being returned to them, with perceived utility/actionability or treatability on such findings being regarded as a major reason. For some, the desire appeared to be related to a veiled sense of ownership and control, and as a means of empowering the concerned people. Some also stated that returning individual results would help promote future research participation. However, some admitted that they would not quarrel with researchers if their research results were not returned but would readily accept them, if disclosed.

“*Research findings help one to know his/her state of wellbeing, there are lots of benefits in returning results to participants such as helping in identifying problem one has and an avenue for rescuing the person. Even though there are risks, the benefits outweigh the risks*” (OW3)

“*For the fact that I participated, I would like to have all my results and make use of them. It is right to return individual results, and the overall results should be returned as well for the benefit of the society. It is a way of appreciating the people and their cooperation. The researchers are obliged to return these results*.” (YM3).

“*Returning the results will encourage others to take part in research but it can also create panic, it is important but should be handled well. Imagine, suddenly receiving news of a bad condition you were not aware of and not having the means of treating it. Or you being told that the condition has no cure. In this case, it may even be better not to know* ”. (YW4)

“*I may not insist on having my individual research results sent to me, but if the researchers do so, I will accept and not complain* ”(OM1)

#### Types of research results or incidental findings to be returned with rationales for their preferences

All the participants desired return of individual results on which a beneficial action could be taken; results which could help them prevent, avoid, control, manage or treat a health condition.

“*Health research is meant to find solutions to health problems; therefore it is good to return any health research result that will provide required solutions to the person’s health problems. Knowledge is power. You can always use the information for one purpose or the other*”. (YW5).

“*If the result will tell me about health conditions which may be present in my family tree, it would be good to know such*”(OM3)

“*Does it really matter if the result is anticipated or incidental? All are results*”(YM3)

“*I am not sure I would like to know of any research result for which I cannot do anything at all about the disease. And this also includes the results which the researcher does not understand*”(OW2)

#### Format and process of returning individual research findings

Nearly all the FGD participants seemed to lean towards a collaborative approach rather than solely relying on researchers or participants. The format for the return of results should be agreed upon with the researchers, considering the anticipated expenses and logistics challenges. While acknowledging the importance of researcher discretion and responsibility, most people would prefer to receive as much actionable information as possible, and preferably in-person from the researcher, with many expressing concerns of confidentiality. The majority would not readily wish to have their results shared with anyone, unless they are allowed to indicate their preferences.

“*The researcher should be ready to find out from me if and how I want my research findings to be communicated to me. If I want it to be shared with anyone, my family setting and other factors will be considered. Otherwise, the results should be disclosed to me face-to-face at the end of the research*.”(OM2)

“*If returning my results to me would cause a heavy burden for the researchers, I may have to forfeit it. Anyway, these things can be worked out as it will not be a bad idea for me to have some information about my health status*”(YW1)

“*Some results, in cases of communicable diseases, may have be revealed to the community but it should be ensured that the person(s) concerned don’t suffer stigmatization*”(OM1)

“*What if the result gets into the wrong hands? Nobody else should be notified of my results, they are personal to me. Such health information is confidential*” YM4

#### Challenges in the return of research findings and measures to mitigate them

The majority of the participants believed that the major challenge would be lack of appropriate funding for the research coupled with the logistics challenges. While most agreed that the sponsors of research should cover all the expenses associated with every aspect of the research, including returning individual results, some suggested that, for certain instances of results dissemination, cost-sharing with the participants could be considered but made voluntary and optional. Other challenges and required measures were suggested.

“*Inadequate fund is a major hindrance in research enterprise*” (YW3)

“*Funding, logistics, negligence of duty, lack of incentive, unfriendly attitude of the participants/community, threat/hostility of the study community, lack of proper policy or its implementation are barriers*” (OW3).

“*Researchers should pay for it, it is their business. Participants have no business with that but only helping the researcher*” (YM1).

“*The participants may pay for the return of important results or confirmation of incidental findings and the government/researchers/sponsors should also help because some participants may not have money to eat ,not to talk of paying for return of research findings*” (OM2)

“*There should be proper policy for the returning of research results, adequate fund should be provided, sensitization is necessary. More training should be provided to researchers on relating with participants and returning results*” (YM4).

## Discussion

Globally, bioethicists have extensively debated the practice of sharing individual anticipated and incidental research findings with study participants. In the global North, there is a growing consensus that participants should have the right to access health-related information from their studies, particularly if the results are clinically useful, meaningful to their health, and can lead to actionable steps(41). The arguments in favor of this position are based on core ethical principles like respect of autonomy, reciprocity, beneficence, nonmaleficence, and transparency. Giving research participants their personal health information, such as health status or genetic predispositions, can empower them to make better-informed decisions about their health, lifestyle, marriage and reproductive choices. However, there are also concerns about sharing such information, including the risk of causing psychological distress, group harm, misinterpreting data, providing results with unclear clinical value, and increasing the workload for researchers. Sharing medical findings without proper counseling or follow-up can cause anxiety, false reassurance, or social stigma. This is worrisome with respect to incidental findings which may not have a clear path for treatment or be understood by society. The practicality of disclosing research findings is a major concern, especially in places like Nigeria. In low- and middle-income countries, the necessary resources for confirmatory testing, continued care, and communicating results in a culturally appropriate way are often scarce. Thus, the main challenge in modern bioethics is finding a balance between what is in the best interest of the participants and what ensures the integrity of the research itself(10, 42).

Taking cognizance of the fact that the perspectives of research participants in Africa on the return of research findings remain largely underexplored, our study set out to determine this within a Nigerian population group. The participants in our study showed good understanding about the basics of health research based on the levels of their self-reported knowledge, despite the perceived low levels of confidence about their knowledge. A strong general desire for the return of both the primary research results and incidental research findings was also expressed among our participants, especially on the premise of clinical utility and actionability/treatability. This is like findings from studies conducted in other parts of the globe(28, 33, 36, 39, 43, 44, 45, 46, 47). Even for results with no clinical utility, 44% of our respondents expressed a desire to have them returned, thereby suggesting the influence of other potential personal reasons for wanting these results. Cultural and social dynamics also play a role. Expectations of solidarity and reciprocity may translate into an obligation on the part of researchers to return selected individual research results in some situations(48, 49). Decisions about health and illness often extend beyond the individual, involving family members, elders, or religious authority figures. Therefore, participants’ expectations about the disclosure of research results may not only reflect personal autonomy but also collective interests(50, 51, 52). Generally, the participants’ perspective favored a decision-making process that leaned towards a shared researcher-participant approach and tended to emphasize the need for community engagement, as well as a process-oriented approach that considers the value of returned results to the participants, risks, and feasibility of return(21, 22, 48). Even with the high level of trust in researchers demonstrated in our study, it appears that the line between health research and healthcare is sometimes blurred from the public perspective(12, 53, 54). A participant might view their enrollment in health research as an opportunity to receive some healthcare benefits (therapeutic misconception). Many Nigerians still rely on out-of-pocket payments for medical care, and access to advanced diagnostics or specialized treatment is limited(55, 56). In this context, participation in health research often carries expectations of tangible benefits, including free diagnostic testing, health information, or access to treatment otherwise unavailable. Such expectations may strongly influence participants’ attitudes toward the return of results. Consequently, participants may place high value on receiving research results, even when incidental. Rather than altruism, their focus is largely on the therapeutic benefit for their personal/family health, which is the hallmark of clinical care. However, the researcher’s primary goal is to gather data, test a hypothesis, and contribute to generalizable knowledge for future patients. This subtle but critical difference in purpose–personal benefit versus scientific advancement– still appears to be a major point of confusion in Nigeria, regardless of a person’s trust in the process(57).

Our findings also indicate that respondents have some difficulty understanding the nuanced differences between primary research results and incidental findings, as demonstrated by their poor knowledge of incidental findings in 87% of the respondents. For most participants, both types of results are health-related, and without specific guidance, it may be hard to grasp why one is the study’s central focus while the other is an unexpected discovery that presents its own set of ethical dilemmas regarding disclosure(58, 59, 60). Ultimately, while an educated and trusting public is a cornerstone of ethical research, these fundamental ambiguities in purpose and discovery remain and need to be addressed, especially for genetics and genomics research.

While various methods of returning results exist, most participants expressed a strong preference for in-person disclosure; a preference rooted in concerns over confidentiality, a fundamental ethical principle that is paramount to protecting a participant’s privacy, providing a safe space to process and understand information that is deeply personal and sensitive, and building trust in the research process. While returning research results by mail or through secure online portals may be more efficient, participants who desire an in-person return of their results are probably motivated by the belief that this method offers the highest level of data security(41). A face-to-face meeting with a researcher or a qualified healthcare provider minimizes the risks associated with electronic transmission, such as data breaches, unauthorized access, or mis-delivery. Moreover, the in-person approach addresses a more human need for a secure and confidential dialogue. Researchers, in turn, can ensure that the participant is the sole recipient of the information and can expectedly address any immediate concerns or distress in a compassionate and confidential manner. This preference challenges researchers to balance efficiency with the ethical imperative to treat participants with the utmost respect and care. On the flip side, it is known that some potential research participants in Nigeria might feel a responsibility to share their health information with their family, and the family might expect to be involved in decisions about their care. Our study demonstrated a strong preference for sharing health-related research information with spouses. The Western concept of individual autonomy may not fully align with the communal values of Africans(61, 62). Therefore, simply returning information to that individual participant, as is often the practice in Western research, may sometimes be inadequate or even inappropriate in such context.

One major challenge to the return of research results which was identified by most of the participants is inadequacy of research funding to cover all aspects of the research. The debate over returning individual research results often hinges on the notion that a widespread policy of returning individual results could strain limited budgets, and that redirecting resources toward this task could potentially slow down the research itself, thus delaying the very discoveries that are meant to benefit the public (3, 4, 33, 41, 63). Cost-sharing between the participants and researchers/research sponsors was suggested by some participants as a pragmatic solution. Participants may be willing to bear a portion of the associated costs, if they want their research results. However, placing the financial burden for return of research results on participants could create an equity problem. It raises issues of fairness and justice for those who could not afford to pay for their results(60, 63, 64). It could exclude individuals from lower socioeconomic backgrounds, who may be the very people who would benefit most from the information. This raises the question of whether access to one’s own health data should be a right or a privilege(65). This tension highlights the ongoing challenge for researchers: to promote a system that not only respects participants’ wishes but also ensures fairness and equity for everyone involved. Proponents of full disclosure argue that the costs of returning results should be factored into the overall research budget from the outset, viewing it as an ethical obligation rather than an optional expense.

Development of appropriate policies and guidelines is another measure recommended by our participants to promote the return of personalized research results. It is contented in some quarters that well-crafted policies and guidelines–rooted in local sociocultural realities and global ethical standards–can bridge the existing gap. Participants in previous studies across the global South frequently recount experiences of never receiving promised test outcomes or finding them so cryptically worded as to be meaningless(66, 67). These firsthand accounts underscore the urgency of policy attention. Before policies can be effective, they must be informed by the specific barriers that participants face. Such challenges include low health literacy, diverse local languages, weak regulatory oversight leading to inconsistencies across studies, financial constraints and inadequate infrastructures(6, 22, 67). Resources to train researchers in collaborative decision-making processes may be needed. The public may need some baseline knowledge to inform their choices about return of primary research results and incidental findings, especially with respect to genetics/genomics research. It is imperative that researchers and research sponsors plan for the return of research results and, more importantly, to include them with greater detail in both research budgets and informed consent forms. By embedding clear consent procedures, investing in capacity building, ensuring cultural sensitivity, strengthening legal protections, promoting confidential safeguards, enshrining community participatory approaches, and committing to continuous evaluation, policymakers, funders, and researchers can honor participants’ rights and bolster the ethical integrity of health research(10, 13, 54, 68)

This study involved potential research participants from one site (Enugu Metropolis), and their responses may not be construed as definitive and generalizable to entire groups in other locations or regions of Nigeria or Africa. Participants in this study were apparently healthy and normal volunteers who were not actively participating in any health research requiring human biological specimens at the time of our study. Their expressed views for hypothetical research situations may not be entirely consistent with expressed views in an actual future research setting, especially for research involving human biological specimens. For the qualitative component of the study, it was challenging to rank or put a number on the importance of some of the opinions that were expressed by the participants. The opinions in such discussions are usually complex and subjective, making it difficult to objectively measure or compare their importance. Because these responses are rich with meaning and nuance, they do not lend themselves to easy numerical ranking. Additional empirical research is thus required in exploring to a greater extent, the identified preferences and opinions expressed in our study.

## Conclusion

The findings underscore strong community demand for the return of individual research results, especially clinically actionable information, but also reveal critical gaps in understanding, resources, and infrastructure. Addressing misconceptions, safeguarding confidentiality, and implementing culturally sensitive, well-funded strategies are essential to ensure ethical and effective return of results in health research. The successful implementation of appropriate policies will depend on collaborative efforts between researchers, policymakers, participants, and communities, focusing on building local capacity, ensuring culturally sensitive communication, and establishing robust, trust-based frameworks which respect the contributions of participants and address their health information needs effectively.

## Supplementary Material

Supplementary Files

This is a list of supplementary files associated with this preprint. Click to download.

• AdditionalFile1QUESTIONNAIRE.docx

• AdditionalFile2FocusGroupDiscussionGuide.docx

## Figures and Tables

**Table 1:** Frequency distribution of 405 participants’ socio-demographic characteristics

Variables N = 405	Frequency	Percentage
**Age categories in years**		
25 and below	93	23.0
26–35	146	36.0
36–45	88	21.7
>45	78	19.3
**Mean (SD)**	**35.46 ± 12.54**	
**Gender**		
Male	204	50.4
Female	201	49.6
**Education**		
No formal education	3	0.7
Primary	18	4.4
Secondary	137	33.8
Tertiary	247	61.0
**Marital status**		
Single	202	49.9
Married	189	46.7
Others (divorced, separated, widowed)	14	3.5
**Employment Status**		
Employed	295	72.8
Unemployed	110	27.2
**Religion**		
Christianity	376	92.8
Others	29	7.2
**Ethnicity**		
Igbo	365	90.1
Others	40	9.9

**Table 2:** Distribution of the respondents with respect to desire for return of the different types of results (RR or IF)

Types of results desired	Yes(%)
Result that is actionable or has clinical utility (preventable, curable, tolerable conditions)	381(94.1)
Result indicates a condition that is clearly curable	371(91.6)
Result indicates a serious condition with late-adult onset of manifestation	276(68.1)
Result that is of no clinical significance/utility	178(44.0)
Result that is not interpretable by the researcher (unknown meaning)	166(41.0)
Result indicates a life-threatening condition	360(88.9)
Result indicates a serious but not life-threatening condition	271(66.9)
Result indicates a genetic, familial or hereditary disease	347(85.7)
Result suggests likelihood of having mental disorder	298(73.6)
Result has potential for stigmatization	270(66.7)
Results suggestive of a risk of developing a condition, regardless of the level of risk	352(86.9%)
Results whose meanings could still change with time	204(50.4%)
Results for non-medical conditions like facial features, skin tone, hair color/texture, fingerprints	182(44.9%)
Results indicating need for further specialized investigations beyond the scope of the research	293(72.3%)

**Table 3:** Distribution of the 405 respondents on the barriers or challenges to the return of research findings.

Reasons cited among the respondents	Frequency (%)
Lack of adequate funding for the research	241(59.5)
Lack of adequate incentives to researchers	83(20.5)
Challenges with logistics	168(41.5)
Ethical concerns	56(13.8)
Regulatory issues	39(9.6)
Researcher lack of interest in return of results	73(18.0)
Low level of health and general literacy of participants	91(22.5)
Participants not desiring the results	124(30.6)
Inconclusive or Incomplete results	148(36.5)
Risk of possible harm to participant or family	89(22.0)
Risk of suspicion, bias or distrust for research/researchers	48(11.9)
Therapeutic misconception by participants	42(10.4)
Fear of results getting into the wrong hands	88(21.7)
Sharing of Aggregate results should suffice	20(4.9)

## Data Availability

The datasets used and analyzed in this study have been presented herein and are available from the corresponding author and first author on request.

## Abbreviations

RRs: Primary Research results
IFs: Incidental findings
HBS: Human Biological Specimen
LGAs: Local Government Areas
FGD: Focus group discussion
YM: Young men
YW: Young women
OM: Old men
OW: Old women
NCHRE: National Code of Health Research Ethics
